# Role of multidrug-resistant bacteria in weaning from invasive mechanical ventilation

**DOI:** 10.1186/s12931-024-02694-5

**Published:** 2024-02-05

**Authors:** Julia D. Michels-Zetsche, Vicky Gassmann, Jasmin K. Jasuja, Benjamin Neetz, Philipp Höger, Jan Meis, Simone Britsch, Urte Sommerwerck, Sebastian Fähndrich, Florian Bornitz, Michael M. Müller, Felix J.F. Herth, Franziska C. Trudzinski

**Affiliations:** 1 Department of Pneumology and Critical Care, Thoraxklinik, University of Heidelberg, Translational Lung Research Center Heidelberg (TLRC-H), Member of the German Center for Lung Research (DZL), Röntgenstrasse 1, D-69126 Heidelberg, Germany; 2 Department for Infectious Diseases, University Hospital Heidelberg, Heidelberg, Germany; 3 Institute of Medical Biometry, University of Heidelberg, Heidelberg, Germany; 4 Department of Cardiology, Angiology, Haemostaseology and Medical Intensive Care, European Center for Angioscience (ECAS), University Medical Center Mannheim, German Center for Cardiovascular Research (DZHK), Partner Site, Heidelberg/Mannheim, Germany; 5 Department of Pneumology, Krankenhaus der Augustinerinnen Cologne, Cologne, Germany; 6 Department of Pneumology, University Medical Center Freiburg, University of Freiburg, Freiburg, Germany; 7 Department of Pneumology and Internal Intensive Care Medicine, Asklepios Hospital Barmbek, Hamburg, Germany

**Keywords:** Multidrug-resistant bacteria, Weaning, Prolonged weaning, Weaning failure, Mortality in weaning

## Abstract

**Background:**

Although multidrug-resistant bacteria (MDR) are common in patients undergoing prolonged weaning, there is little data on their impact on weaning and patient outcomes.

**Methods:**

This is a retrospective analysis of consecutive patients who underwent prolonged weaning and were at a university weaning centre from January 2018 to December 2020. The influence of MDR colonisation and infection on weaning success (category 3a and 3b), successful prolonged weaning from invasive mechanical ventilation (IMV) with or without the need for non-invasive ventilation (NIV) compared with category 3c (weaning failure 3cI or death 3cII) was investigated. The pathogen groups considered were: multidrug-resistant gram-negative bacteria (MDRGN), methicillin-resistant *Staphylococcus aureus* (MRSA) and vancomycin-resistant *Enterococcus spp.* (VRE).

**Results:**

A total of 206 patients were studied, of whom 91 (44.2%) showed evidence of MDR bacteria (32% VRE, 1.5% MRSA and 16% MDRGN), with 25 patients also meeting the criteria for MDR infection. 70.9% of the 206 patients were successfully weaned from IMV, 8.7% died. In 72.2% of cases, nosocomial pneumonia and other infections were the main cause of death. Patients with evidence of MDR (infection and colonisation) had a higher incidence of weaning failure than those without evidence of MDR (48% vs. 34.8% vs. 21.7%). In multivariate analyses, MDR infection (OR 4.9, *p* = 0.004) was an independent risk factor for weaning failure, along with male sex (OR 2.3, *p* = 0.025), Charlson Comorbidity Index (OR 1.2, *p* = 0.027), pH (OR 2.7, *p* < 0.001) and duration of IMV before admission (OR 1.01, *p* < 0.001). In addition, MDR infection was the only independent risk factor for death (category 3cII), (OR 6.66, *p* = 0.007).

**Conclusion:**

Patients with MDR infection are significantly more likely to die during the weaning process. There is an urgent need to develop non-antibiotic approaches for the prevention and treatment of MDR infections as well as clinical research on antibiotic stewardship in prolonged weaning as well as in ICUs.

## Background

Patients who present to a specialist weaning centre for weaning from invasive mechanical ventilation (IMV) are a very heterogeneous group of patients: regardless of what ultimately led to the initiation of invasive ventilation, these patients often suffer from pre-existing cardiac and pulmonary disease and have often had long stays in acute intensive care units [1]. Multi-drug resistant bacteria (MDR) represent an increasing burden in this population [2]. The reasons for this are multi-layered. Firstly, patients have often received a large number of antibiotics during their hospital stay, putting them at high risk of developing antibiotic resistance [2, 3]. Other risk factors include multimorbidity, previous hospitalization and often impaired lung function, which in itself is a risk factor for colonisation or infection with multidrug resistant gram-negative bacteria (MDRGN) [4–6]. The most recent and largest study on weaning “WEAN SAFE”, an international, multicentre, prospective observational cohort study enrolled 5869 patients in 481 intensive care units (ICUs). The identified risk factors for weaning failure were as in previous studies i.e. older age, comorbidities, frailty, degree of respiratory dysfunction but also deep sedation and delay in separation attempt [7].

However, multi-drug resistant pathogens, which are often a major challenge in the daily routine of weaning centres, have received little attention in the context of ventilator weaning, although there is data showing that MDR bacteria colonisation is a hinderance in respiratory and neurological rehabilitation [8, 9]. Group therapy and participation in certain rehabilitation activities such as physiotherapy, speech therapy and respiratory therapy are more difficult because of the need for patient isolation due to this colonisation. However, these measures all play a crucial role in the weaning process [10]. It is therefore likely that limited access to these resources is associated with an increased risk of weaning failure. In addition, the choice of antibiotics for acute infections, which are often a step backwards in the weaning process, is limited, and it is likely that patients with MDR pathogens will require more frequent changes of antibiotic and prolonged antibiotic administration during the course of treatment [11]. Whether this also has implications for the weaning process is not well understood.

The aim of the current study was therefore to investigate the effects of MDR colonisation and infection in patients undergoing prolonged weaning in a large university hospital weaning centre.

## Methods

### Study design and participants

This study is a retrospective analysis of consecutive patients from one university centre for ARDS patients and prolonged weaning. From a computerised database, we reviewed all patients who received invasive ventilation in our intensive care unit (ICU) or ventilator weaning unit (VWU) from January 2018 to December 2020. All patients who met the criteria for prolonged weaning, defined by the International Consensus Conference (ICC) held in April 2005 as weaning group 3 (patients who fail at least 3 weaning attempts, or require 7 days of weaning after the first SBT), were included in the analysis, [12]. Patients from ICC group 1 (simple weaning: patients who proceed from initiation of weaning to successful extubation on the first attempt without difficulty) and 2 (difficult weaning: patients who fail the initial weaning attempt and require up to 3 SBTs, or as long as 7 days from the first SBT to achieve successful weaning) were excluded as well as patients with ECMO (extracorporeal membrane oxygenation) and patients without any weaning attempt. Weaning outcome was classified according to the German national guidelines as successful prolonged weaning from IMV without the need for subsequent long-term NIV, category (3a), successful prolonged weaning from IMV with continuation of NIV (3b) and failed weaning from IMV (3c), the last group including patients who continued IMV in an outpatient setting (3c I) and those who died during the weaning process (3c II) [10]. No relevant data was missing.

For the retrospective data analysis, a vote of the Ethics Committee of the University of Heidelberg (No. S-760/2020) was obtained.

### Weaning process

Most patients requiring IMV were admitted from external hospitals after resolution of acute critical illness. Others required long-term invasive ventilation after thoracic surgical treatment or during the course of their stay at our ICUs after resolution of acute critical illness corresponding to the transferred patients. A structured weaning process based on the German guideline on prolonged weaning is implemented in both the ICU and the VWU [10, 13]. Once the acute critical illness had resolved and clinical stability was sufficient, patients on prolonged weaning were transferred internally from the ICU to the VWU. The initial diagnostic examination served to identify the cause of the ventilator dependence, such as respiratory muscle fatigue or weakness, increased load on the respiratory muscles, altered respiratory drive or metabolic aspects. Weaning readiness was assessed using clinical and objective criteria adapted from Boles 2007 [12]. Knowing well that not all criteria have to be met to start weaning, a liberal approach was applied so that the first spontaneous breathing trial (SBT) was performed as early as possible after admission. If the patient was not ready for weaning, the underlying acute problem was addressed, e.g. reduction of sedation or treating acute infections. In all patients enrolled, the SBT were performed with low pressure support or on T-piece/speaking valve with the latter approach being preferred to the former in tracheostomised patients [14].

Between consecutive SBTs, the respiratory muscles were unloaded with assist-control ventilation to avoid load-capacity imbalance and to allow muscle regeneration. During the course, SBTs were prolonged, which allowed reconditioning of the respiratory muscles and, in the best case, allowed for successful weaning from the ventilator. The weaning process was accompanied by a multidisciplinary team consisting of respiratory therapists, physiotherapists, specialized nurses, speech therapists and psychologists. Their core tasks included tracheal cannula management, therapy of dynamic hyperinflation, secretion management, dysphagia management and daily mobilization. Following the weaning process, the transition to an appropriate health care facility was ensured prior to discharge.

### Laboratory tests

According to our institutional standards, blood values were measured daily, usually in the morning. Blood gas analysis (BGA) was taken several times a day depending on the former BGA and ventilator settings.

For our analysis, data were collected from each patient at three time points: the first weaning attempt, seven days after the first SBT and at discharge, which for deceased patients was the death date. If no data was available at these time points, the values that where chronologically closest were used. For patients who were discharged on day seven, we used the same blood values for time point two and time point three. The analysed blood values were haemoglobin (Hb), C-reactive protein (CRP), blood urea nitrogen (BUN), creatinine and estimated glomerular filtration rate (eGFR) [15]. The analysed BGA values were pH, partial pressure of oxygen in the arterial blood (PaO_2_), partial pressure of carbon dioxide in the arterial blood (PaCO_2_), and base excess (BE).

### MDR definition

For the current study, multidrug-resistant gram-negative bacteria (MDRGN) were defined as carbapenem resistant Enterobacterales (CRE) with resistant minimal inhibitory concentration (MIC) or molecular detection of carbapenemases (4 MDRGN: Gram negative bacteria resistant to all 4 antibiotic groups). Additionally, Enterobacterales and *Pseudomonas aeruginosa* with simultaneous resistance to piperacillin/tazobactam, ceftazidime/ceftriaxone/cefotaxime and ciprofloxacin but susceptibility to carbapenems were included (3 MDRGN: MDRGN resistant to 3 antibiotic groups).

All methicillin-resistant *Staphylococcus aureus* (MRSA) isolates with detected resistance to methicillin were included.

All *Enterococcus faecium (E. faecium)* and *Enterococcus faecalis (E. faecalis)* isolates with detectable vancomycin resistance (VRE) were included.

### Colonisation and infection

The differentiation between colonisation and infection is very difficult. The patients with detection of MDR were analysed for infection due to the physicians’ reports, radiological findings and CRP levels. The focus of the infection, the location of the detection of MDR and the antibiotic therapy used were also taken into account. If another pathogen was found at the site of infection and/or the patient recovered with an antibiotic therapy to which the MDR found was resistant, it was concluded that the MDR was not the cause of the infection.

### Microbiological diagnostics

According to an institutionally standardised protocol, screening for VRE and MDRGN by rectal swab and for MRSA by nasal swab was performed regularly on admission. Tracheal or bronchial aspirates were taken if a respiratory tract infection was suspected, or as part of a general search for infectious foci if deemed necessary. The collected samples were sent by courier to the Laboratory for Medical Microbiology at the University of Heidelberg.

For screening purpose, rectal swabs were subcultured on blood agar (Columbia agar, 5% sheep blood, BD) as growth control, *S. aureus*/MRSA biplate (BD BBLTM CHROMagarTM Staph aureus/BBLTM CHROMagarTM MRSA II, BD), chromID® VRE (bioMérieux) and ESBL Chromagar (bioMérieux). The swabs were processed in the fully automatic part of our Total Lab Automation (TLA, BD Kiestra™). Imaging was automatically done after 20 h of incubation, except the chromID® VRE agar which was imaged after 36 h-incubation.

Suspicious pink colonies on the MRSA side were confirmed by molecular detection of mecA/mecC gene. Colonies on chromID VRE were first identified with MALDI-TOF MS (Microflex and Smart, Bruker Daltonik GmbH, Bremen) for *E. faecium* and *E. faecalis*, respectively. In case of first evidence of VRE, resistance gene was confirmed by PCR (vanA/vanB). Growth on ESBL agar was identified with MALDI-TOF MS and tested with VITEK2 (bioMérieux) to evaluate MDRGN.

Speciments from the respiratory tract were manually subcultured on blood agar, chocolate agar (bioMérieux), MacConkey agar (bioMérieux) and Candida BBL™ CHROMagar™ Orientation (BD). In the case of bronchoalveolar lavage (BAL) a selective agar for hyphomycetes and Schaedler/KV agar (5% sheep blood, BD) for anaerobics was added. *S. aureus* suspicious colonies on blood agar were tested on catalase and coagulase, identified with MALDI-TOF MS and tested with VITEK2. Additionally, subculturing on *S.aureus*/MRSA biplate was done. Colonies on MacConkey were processed via MALDI-TOF MS and VITEK2. In case of carbapenem resistance, molecular detection was added.

Enterococcus species were visually identified and not tested, due to lack of clinical significance in respiratory tract.

### Statistical analysis

Statistical analysis was performed using SPSS version 28 (SPSS Inc., Chicago, Ill., USA). Numerical variables were analysed using Student’s t-test, or ANOVA (Analysis Of Variance),Mann-Whitney U-test or Kruskal-Wallis-Test as appropriate. For categorical variables, group comparisons were made using the chi-square test comparing MDR infection, MDR colonisation and without detection of MDR (non-MDR) patients. Results were considered statistically significant at p values < 0.05. All significant variables were analysed using binary logistic regression analysis to assess independent risk factors for weaning failure and death.

## Results

A total of 206 patients, 45.1% female and 54.9% male, were analysed. The majority of patients were intubated for acute respiratory failure, with only 15.0% intubated for elective surgery. MDR colonisation or infection was present in 91 cases (44.2%). The majority of MDR cases (66) were VRE, 33 patients had an infection/colonisation with MDRGN, and MRSA was detected in 3 patients. 11 patients had double infection/colonisation with VRE and MDRGN and 1 with VRE and MRSA. MDR were mainly detected by rectal swabs in 78 cases or by tracheal or bronchial aspirates in 23 cases. 92.3% of MDR positive patients had an infection and 72.5% of the MDR positive patients had a lower respiratory tract infection at least once since admission. Only 27.5% of the patients with detected MDR had an infection caused by MDR as defined above, concluding that only 29.8% of the infections in MDR positive patients were caused by the detected MDR. The characteristics of the patients at the time of admission and the results of the microbial diagnostics are listed in Table 1.

**Table 1:** Patient characteristics

Anthropometric data	Mean ± STABW
Sex female	93/206 (45.1%)
Sex male	113/206 (54.9%)
Age (years)	65.0 ± 13.1
**Cause of IMV**	
- Acute respiratory failure	175/206 (85.0%)
- postoperative	31/206 (15.0%)
**Duration of IMV**	
- before admission (d)	51.7 ± 171.5
- before the first SBT (d)	55.2 ± 170.9
**Multidrug-resistant bacteria**	
Colonisation/infection with multidrug-resistant bacteria	91/206 (44.1%)
Methicillin-resistant Staphylococcus aureus (MRSA)	3/206 (1.5%)
Vancomycin-resistant Enterococcus spp. (VRE)	66/206 (32%)
Multidrug-resistant gram-negative (MDRGN) bacteria	33/206 (16%)
*- Pseudomonas aeruginosa*	23/206 (11.2%)
*- Acinetobacter baumanii*	1/206 (0.5%)
*- Enterobacter cloacae complex*	6/206 (2.9%)
*- Morganella morganii*	1/206 (0.5%)
*- Escherichia coli*	1/206 (0.5%)
*- Klebsiella aerogenes*	2/206 (1%)
*- Klebsiella oxytoca*	1/206 (0.5%)
**Isolation of pathogens**	
- rectal swab	77 (37.4%)
- other skin swabs	2 (1%)
- wound fluid/drains	12 (5.8%)
- tracheal or bronchial aspirate	22 (10.7%)
- blood culture central/peripheral	2 (1%)
- catheter urine	13 (6.3%)

Abbreviations: IMV (invasive mechanical ventilation); MDRGN (Multidrug-resistant gram-negative bacteria); MRSA (Methicillin-resistant Staphylococcus aureus); SBT (spontaneous breathing trial); VRE (Vancomycin-resistant Enterococcus spp)

The comparison of patients with MDR infection with MDR colonisation and without MDR detection (non-MDR) showed a significantly longer duration in days of IMV before admission to the weaning centre (67.9 vs. 48.3 vs. 50.1, *p* = 0.006) in patients with MDR infection. The length of stay (LOS) in days was significantly different as well (*p* = 0.012) and longest in MDR infections (62,48 ± 25,00). Between MDR colonisation (48.42 ± 31.31) and non-MDR (41.55 ± 32.10) was no significant difference (*p* = 0.16), Table 2. Non-MDR patients were less likely to be postoperative and more likely to have COPD. CRP was significantly different with highest levels in MDR infection followed by MDR colonisation and lowest in non-MDR at the time of the first SBT, as well as at the last measurement. The comparison of the two groups is shown in Table 3.

**Table 2:** Patient characteristics stratified for MDR colonisation/infection

	all patients *N* = 206	MDR not detected *N* = 115	MDR colonisation *N* = 66	MDR infection *N* = 25	***p***-value
**Anthropometric data**					
Sex female	93 (45.1%)	57 (49.6%)	28 (42.4%)	8 (32.0%)	0.241
Sex Male	113 (54.9%)	58 (50.4%)	38 (57.6%)	17 (68.0%)	-
Age	65.0 ± 13.1	64.6 ± 13.2	66.8 ± 13.1	61.8 ± 12.4	0.247
IMV before admission (d)	51.6 ± 171.5	50.1 ± 215.6	48.3 ± 67.0	67.9 ± 134.2	**0.006**
IMV before the first SBT (d)	55.2 ± 170.9	53.8 ± 215.1	51.3 ± 65.7	71.4 ± 133.3	**0.005**
**Admission diagnoses and comorbidity index**					
ARDS	59 (28.6%)	31 (27%)	23 (34.8%)	5 (20%)	0.314
NMD	10 (4.9%)	7 (6.1%)	3 (4.5%)	0 (0%)	0.434
postoperative	31 (15.0%)	10 (8.7%)	14 (21.2%)	7 (28.0%)	**0.012**
COVID-19	9 (4.4%)	3 (2.6%)	3 (4.5%)	3 (12.0%)	0.114
COPD	68 (33%)	48 (41.7%)	14 (21.2%)	6 (24.0%)	**0.011**
Others	29 (14.1%)	16 (13.9%)	9 (13.6%)	4 (16.0%)	0.956
CCI ^§^	4.5 ± 2.2	4.4 ± 2.3	4.7 ± 1.8	4.4 ± 1.9	0.613
Respirator settings *					
MV (L)	8.3 ± 3.2	9.2 ± 3.4	8.7 ± 3.1	8.4 ± 2.8	0.610
Pmax** (cmH_2_0)	15.5 ± 4.1	15.5 ± 4.4	15.5 ± 4.0	15.5 ± 3.4	0.994
PEEP (cmH_2_0)	7.1 ± 2.6	7.2 ± 2.7	7.1 ± 2.3	6.7 ± 2.7	0.610
FiO_2_%	32.7 ± 13.3	33.2 ± 12.5	30.3 ± 12.0	36.6 ± 18.7	0.128
**Blood gases**					
SaO_2_(%) arterial BGA	95.4 ± 3.7	95.7 ± 3.2	94.7 ± 4.7	96.2 ± 2.3	0.103
PaO_2_ (mmHg)	84.4 ± 23.5	86.3 ± 25.7	78.9 ± 19.0	89.8 ± 21.5	0.057
PaCO_2_ (mmHg)	44.8 ± 11.1	44.5 ± 9.8	44.2 ± 11.9	48.3 ± 14.0	0.257
BE	4.7 ± 4.7	5.4 ± 4.3	4.0 ± 4.9	4.0 ± 5.7	0.118
pH	7.43 ± 0.07	7.44 ± 0.06	7.43 ± 0.08	7.43 ± 0.06	0.053
**Laboratory values**					
Hb* (g/dL)	9.4 ± 1.5	9.7 ± 1.5	9.3 ± 1.5	9.05 ± 1.07	0.066
CRP* (mg/L)	80.2 ± 72.9	69.2 ± 64.0	85.6 ± 78.9	115 ± 84	**0.012**
CRP (d7)	60.4 ± 60.8	53.4 ± 53.2	65.2 ± 72.3	79.7 ± 56.2	0.108
CRP (at discharge/last CRP)	40.4 ± 63.2	32.3 ± 57.8	39.7 ± 63.6	79.3 ± 73.2	**0.003**
Creatinine*	1.1 ± 0.9	0.9 ± 0.8	1.2 ± 1.2	1.2 ± 1.0	0.194
BUN*	67.2 ± 44.9	65.7 ± 48.1	65.59 ± 39.4	78.6 ± 43.5	0.406
eGFR*(ml/min/1,73 m²)	84.4 ± 43.1	87.9 ± 45.2	80.8 ± 41.7	78.7 ± 37.4	0.452

The *p*-values refer to the comparison between the three groups „MDR not detected“, „MDR infection“, „MDR colonisation“

*during first weaning trial, **above PEEP, ^§^ [29]

Abbreviations: ARDS (acute respiratory distress syndrome); BE (base excess); BGA (blood gas analysis); BUN (blood urea nitrogen); CCI (Charlson comorbidity index); COPD (chronic obstructive pulmonary disease); COVID-19 (Corona virus disease 2019); CRP (C-reactive protein); eGFR (estimated glomerular filtration rate); Hb (haemoglobin); IMV (inasvie mechanical ventilation); MDR (multidrug-resistant bacteria); MV (minute ventilation); N (number) NMD (neuromuscular disease); PaCO_2_ (arterial pressure of carbon dioxide); PaO_2_ (arterial pressure of oxygen); PEEP (positive end expiratory pressure); pH (potential of hydrogen); Pmax (maximal ventilation pressure above PEEP); *p*-value (probability); SaO_2_ (arterial saturation of oxygen); SBT (spontaneous breathing trial)

**Table 3:** Weaning outcomes, stratified for MDR colonisation/infection

Variables	all patients *N* = 206	MDR not detected *N* = 115	MDR colonisation *N* = 66	MDR infection *N* = 25	***p***-value
LOS weaning centre (days)	46.3 ± 32.9	41.55 ± 32.10	48.42 ± 31.31	62,48 ± 25,00	**0.012**
**Weaning categories**					
***3a.*** **Successful prolonged weaning from IMV without the need for subsequent long-term NIV**					
− 3a I extubation/decannulation	66 (32.0%)	36 (31.3%)	20 (30.3%)	10 (40%)	0.655
− 3a II without decannulation	9 (4.4%)	6 (5.2%)	3 (4.5%)	0 (0%)	0.510
**3b. Successful prolonged weaning from IMV with the continuation of NIV**					
− 3b I without nursing care	25 (12.1%)	18 (15.7%)	6 (9.1%)	1 (4%)	0.177
− 3b II with additional nursing care	46 (22.3%)	30 (26.1%)	14 (21.2%)	2 (8%)	0.139
**3c. Failed weaning from invasive IMV**					
− 3c I continued IMV	42 (20.4%)	20 (17.4%)	16 (24.2%)	6 (24%)	0.486
− 3c II death	18 (8.7%)	5 (4.3%)	7 (10.6%)	6 (24%)	**0.006**
**Weaning failure (3c)**					
	60 (29.1%)	25 (21.7%)	23 (34.8%)	12 (48%)	**0.015**

Subgroups of prolonged weaning patients according to the current German guideline’s definition [10]

Abbreviations: IMV (invasive mechanical ventilation); LOS (length of stay); MDR (multidrug-resistant bacteria); N (number); NIV (non-invasive ventilation); *p*-value (probability)

Out of a total of 206 patients, 70.9% were successfully weaned from invasive ventilation. There was a significant difference in weaning failure (category 3c) between the three groups, with highest rate in MDR infection (48%) followed by MDR colonisation (34.8%) and lowest in non-MDR (21.7%), *p* = 0.015. This was mainly due to the difference in death as outcome (3cII) with MDR infection 24%, MDR colonisation 10.6% and non-MDR 4.3%, *p* = 0.006.

In multivariate analyses, MDR infection (OR 4.91, *p* = 0.004) was an independent risk factor for weaning failure, along with male sex (OR 2.29, *p* = 0.025), Charlson Comorbidity Index (OR 1.24 for each additional point, *p* = 0.027), pH at first SBT (OR 2.68 for each 0.1 change, *p* = < 0.001) and duration of IMV before admission in days (OR 1.01, *p* < 0.001), Table 4. MDR colonisation was no significant predictor of weaning failure but showed a trend towards it (OR 1.91, *p* = 0.092).

**Table 4:** Weaning failure category 3c I and 3c II multivariate analysis

Predictors	B	SE	OR	95%CI for OR		***p***-value
				Lower	Upper	
**Age (y)**	-0.01	0.02	0.99	0.96	1.03	0.753
**Sex (f/m)**	0.83	0.37	2.29	1.11	4.73	**0.025**
**CCI (points)**	0.22	0.10	1.24	1.02	1.50	**0.027**
**IMV before admission (days)**	0.01	0.00	1.01	1.00	1.02	**< 0.001**
**pH (0.1)**	0.99	0.31	2.68	1.47	4.88	**< 0.001**
**MDR infection**	1.59	0.55	4.91	1.67	14.43	**0.004**
**MDR colonisation**	0.65	0.38	1.91	0.90	4.05	0.092

Abbreviations: B (unstandardised estimate); CI (confidence interval); CCI (Charlson comorbidity index); IMV (inasvie mechanical ventilation); MDR (multidrug-resistant bacteria); OR (odds ratio); pH (potential of hydrogen); p-value (probability); SE (standard error)

A total of 18 patients (8,7%) died. Of these 13 (72.2%) died of infection, without a significant difference between MDR (69.2%) and non-MDR patients (80.0%). Deadly infections included 8 ventilator acquired pneumonias (VAP), 3 infections/sepsis with unknown focus, 1 abdominal infection, 1 phlegmon of the lower limb. Other causes of death included 3 patients who died of respiratory failure, all in a palliative setting, 1 of right heart failure, 1 of colonic ischaemia.

In multivariate logistic regression analyses, MDR infection was the only independent risk factor for death (category 3c II), (OR 6.66, *p* = 0.007), Table 5. A trend for male gender (OR 2.74, *p* = 0.093) and MDR colonisation (OR 2.47, *p* = 0.147) could be seen.

**Table 5:** Death, weaning category 3c II multivariate analysis

Predictors	B	SE	OR	95%CI for OR		***p***-value
				Lower	Upper	
**Age (y)**	0.00	0.02	1.00	0.95	1.04	0.885
**Sex (f/m)**	1.01	0.60	2.74	0.84	8.90	0.093
**CCI (points)**	0.18	0.14	1.19	0.90	1.59	0.220
**IMV before admission (days)**	0.00	0.00	1.00	1.00	1.00	0.544
**pH (0.1)**	0.01	0.38	1.01	0.47	2.13	0.989
**MDR infection**	1.90	0.70	6.66	1.70	26.09	**0.007**
**MDR colonisation**	0.90	0.62	2.47	0.73	8.40	0.147

Abbreviations: B (unstandardised estimate); CCI (Charlson comorbidity index); IMV (inasvie mechanical ventilation); MDR (multidrug-resistant bacteria); OR (odds ratio); pH (potential of hydrogen); *p*-value (probability); SE (standard error)

## Discussion

The aim of our analysis was to investigate the impact of MDR colonisation and infection on weaning success in patients undergoing prolonged weaning. Our main findings are that MDR colonisation is common in patients transferred to a weaning centre. In the patient population we studied, 44.2% of cases were colonised (32.0%) or infected (12.1%) with one or more of the MDR groups (MRSA, VRE or 3/ 4 MDRGN bacteria resistant to 3 or all 4 antibiotic groups respectively). According to the current national weaning guideline “prolonged weaning”, weaning failure category 3c is present if the patient (I) is discharged to outpatient care with continued invasive ventilation or (II) dies in the weaning centre. In the total population, weaning failure category 3c occurs in 29.1% of cases with a significant difference between non-MDR, MDR colonisation and MDR infection with an increase in that order.

MDR infection was an independent risk factor for weaning failure in the multivariate analyses, even after adjusting for age, sex, duration of previous mechanical ventilation and the sum of comorbidities. There was a trend for MDR colonisation. The absolute proportion of MDR infection patients discharged to invasive home mechanical ventilation was 48% and 34.8% for MDR colonisation, and the majority of deceased patients had an MDR infection or colonisation (72.2%). MDR infection was also an independent risk factor for mortality in the weaning centre (OR 6.66). Even after adjustment for co-morbidities, age and sex, additional factors such as underlying disease severity, which may not be fully reflected here, and general poorer health certainly play a role in MDR infection. The fact that MDR infected patients had elevated CRP levels on admission and during the course of therapy, and that the causes of death were almost all due to nosocomial infections, suggests that incurable infections contributed to this poor outcome, although only 29.8% of the infections in MDR positive patients were caused by MDR.

A meta-analysis of 21 studies of patients with MDRGN and non-MDRGN infections showed that the presence of MDRGN and inadequate treatment of MDRGN were predictors of mortality [16]. Patients undergoing prolonged weaning are particularly at risk of MDR colonisation because, in addition to mechanical ventilation, they often have a history of prolonged ICU stays, the presence of invasive devices and the use of broad-spectrum antibiotics, all of which place them at high risk of infection with MDR pathogens [17–20]. In our analyses, we also considered the colonisation of the rectum with VRE, as this is also a risk factor for MDR infections [21]. The distribution of MDR in our population, with most isolates from the rectum, followed by the respiratory tract and urine, was similar to other studies [22], although respiratory findings are more common in our population, probably due to the nature of a weaning ward. Accordingly, nosocomial pneumonia was the main cause of death in our study.

The findings in our study that non-MDR patients were less likely to be postoperative and more likely to have COPD was surprising. We do not have an explanation.

Patients with MDR infection were significantly longer ventilated before transfer to the weaning centre and had a significantly longer LOS in the weaning centre without a difference between MDR colonisation and non-MDR. The complex management of these patients is not only time consuming. It is also staff intensive. It is surprising that this problem has remained largely unaddressed in the literature, given that MDR infection has an impact on the prognosis of these patients and that expensive, complex therapy is often unsuccessful in this population.

Only one study to date, by Bickenbach et al., examined the effect of infection with MDR on weaning outcome and showed a negative effect on weaning outcome, comparing 3a and 3c, as outcome 3b was not included in the analysis. There was no significant effect on mortality. The prevalence of MDR infections was 24.8%, including 20% MRSA and 80% 3 MDRGN/4 MDRGN [23]. The difference of their study is that they examined patients from an acute ICU, so the number of patients in weaning categories 3a was higher than in our population, which consisted of patients examined after transfer to a weaning centre due to the need for prolonged weaning. Furthermore, the length of stay (LOS) was longer in our collective than in the one of Bickenbach et al. (46.3 ± 32.9 vs. 23.2 ± 21.7days). In that study the colonisation increased with the LOS, which might explain our higher detection rate of MDR (44.1% vs. 24.8%). Both points could be a reason why MDR infection was not a risk factor for mortality in their study. Nevertheless, in our study the LOS was longer for patients with MDR infection, but it was similar between non-MDR and MDR colonisation. Thus, we conclude that MDR colonisation does not increase with LOS but that patients with MDR infections have a longer LOS due to the time needed to treat these infections and their complications.

The missing effect of MDR infection on mortality in the Bickenbach et al. study is surprising and contrasts with other studies, such as the analysis by Magret et al., which showed that nosocomial bacterial pneumonia was more common in patients with MRSA and *A. baumannii* colonisation and prolonged mechanical ventilation, and was independently associated with higher mortality [24], or Agbaht et al., who showed that bacteraemic ventilator-associated pneumonia in previously hospitalised patients was more often caused by MRSA and was independently associated with increased ICU mortality [25]. Considering the different pathogens, resistance and colonisation patterns, colonisation with MDR should be considered in a more differentiated way. In a surveillance study of 147 Spanish ICUs, colonisation or infection with different MDR pathogens and prolonged care were independent risk factors for MRSA colonisation [26]. In general, the exponential rise in antimicrobial resistance among virulent pathogens is currently one of the greatest challenges for clinicians in the ICU. Antimicrobial stewardship is a multi-faceted approach that aims to combat the emergence of antibiotic resistance, improve patient outcomes and control healthcare costs by optimising the use of antimicrobial agents [27]. Antimicrobial stewardship and knowledge of local epidemiology, patient risk stratification and infection control measures remain key elements in the management of MDR infections. As a last resort in the treatment of complex intensive care patients, weaning centres are primarily dependent on the acute intensive care units, where the above measures must also be introduced and consistently followed and implemented with regard to the overall prognosis [28].

There is a great need for clinical research, particularly in the area of weaning, as shown by the largest weaning study, the WEAN SAFE study. Of the 5869 critically ill patients enrolled in the study who received IMV for at least 2 days, only 65% could be weaned at 90 days. Factors associated with risk included age, immunodeficiency and frailty score, cardiac arrest or non-traumatic neurological event as the reason for ICU admission, pre-existing care limitations and the degree of respiratory compromise and ventilatory support used at the time of the first weaning attempt; the presence of deep sedation levels at the time of the first weaning attempt was associated with weaning failure; and the time interval between the establishment of weaning criteria and the first weaning attempt. There is no information about the patients MDR status given [7].

### Limitations

The main limitations are that this is a monocentre and retrospective study. Our study population consists of 206 patients, which is rather small compared to other weaning studies. Nevertheless, it is the first study to analyse the effect of MDR infection and colonisation on weaning outcomes in patients with prolonged weaning, an important and undervalued topic. Furthermore, the effect of MDR infection on mortality is rather overwhelming.

## Conclusion

Patients with MDR infection are significantly more likely to die during the weaning process. We believe that the increasing prevalence of MDR bacterial infections in critically ill patients needs to be addressed to improve weaning and prolonged weaning outcomes. In this regard, there is an urgent need to develop non-antibiotic approaches for the prevention and treatment of MDR infections in prolonged weaning and the ICU. Finally, clinical research demonstrating the benefits of antimicrobial stewardship in ICUs and specialised weaning centres is essential.

**Tables**.

## Data Availability

The datasets used and analysed during the current study are available from the corresponding author on reasonable request.

## Declarations

### Take home message

Patients with MDR infection in prolonged weaning are significantly more likely to have weaning failure or die.

## Abbreviations

ARDS: acute respiratory distress syndrome
B: unstandardized estimate
BE: base excess
BGA: blood gas analysis
BUN: blood urea nitrogen
CCI: Charlson comorbidity index
CI: confidence interval
CIM: critical illness associated myopathy
CIP: critical illness associated polyneuropathy
COPD: chronic obstructive pulmonary disease
COVID-19: Corona virus disease 2019
CRP: C-reactive protein
eGFR: estimated glomerular filtration rate
FiO_2_: inspiratory fraction of oxygen
Hb: haemoglobin
ICC: International Consensus Conference
ICU: intensive care unit
IMV: invasive mechanical ventilation
LOS: length of stay
MDR: multidrug-resistant bacteria
MDRGN: multidrug-resistant gram-negative bacteria
MRSA: methicillin-resistant Staphylococcus aureus
MV: minute ventilation
NIV: non-invasive ventilation
NMD: neuromuscular disease
OR: odds ratio
PaCO_2_: arterial pressure of carbon dioxide
PaO_2_: arterial pressure of oxygen
PEEP: positive end expiratory pressure
pH: potential of hydrogen
Pmax: maximal ventilation pressure above PEEP
RASS: Richmond Agitation-Sedation Scale
RR: respiratory rate
SaO_2_: arterial saturation of oxygen
SBT: spontaneous breathing trial
SE: standard error
VAP: ventilator acquired pneumonia
VRE: vancomycin-resistant Enterococcus spp
V_T_ tidal: volume
VWU: ventilator weaning unit

## References

[1] WeanNet Study G (2016). [WeanNet: the network of weaning units of the DGP (Deutsche Gesellschaft fur pneumologie und Beatmungsmedizin) - results to epidemiology an outcome in patients with prolonged weaning]. Dtsch Med Wochenschr.

[2] Timsit JF, Bassetti M, Cremer O, Daikos G, de Waele J, Kallil A, Kipnis E, Kollef M, Laupland K, Paiva JA, Rodriguez-Bano J, Ruppe E, Salluh J, Taccone FS, Weiss E, Barbier F (2019). Rationalizing antimicrobial therapy in the ICU: a narrative review. Intensive Care Med.

[3] Tacconelli E, Gorska A, De Angelis G, Lammens C, Restuccia G, Schrenzel J, Huson DH, Carevic B, Preotescu L, Carmeli Y, Kazma M, Spanu T, Carrara E, Malhotra-Kumar S, Gladstone BP (2020). Estimating the association between antibiotic exposure and colonization with extended-spectrum beta-lactamase-producing Gram-negative bacteria using machine learning methods: a multicentre, prospective cohort study. Clin Microbiol Infect.

[4] Dantas LF, Dalmas B, Andrade RM, Hamacher S, Bozza FA (2019). Predicting acquisition of carbapenem-resistant Gram-negative pathogens in intensive care units. J Hosp Infect.

[5] Pop-Vicas A, Mitchell SL, Kandel R, Schreiber R, D’Agata EM (2008). Multidrug-resistant gram-negative bacteria in a long-term care facility: prevalence and risk factors. J Am Geriatr Soc.

[6] Karanika S, Karantanos T, Arvanitis M, Grigoras C, Mylonakis E (2016). Fecal colonization with extended-spectrum beta-lactamase-producing Enterobacteriaceae and risk factors among healthy individuals: a systematic review and metaanalysis. Clin Infect Dis.

[7] Pham T, Heunks L, Bellani G, Madotto F, Aragao I, Beduneau G, Goligher EC, Grasselli G, Laake JH, Mancebo J, Penuelas O, Piquilloud L, Pesenti A, Wunsch H, van Haren F, Brochard L, Laffey JG, Investigators WS (2023). Weaning from mechanical ventilation in intensive care units across 50 countries (WEAN SAFE): a multicentre, prospective, observational cohort study. Lancet Respir Med.

[8] Vitacca M, Marino S, Comini L, Fezzardi L, Paneroni M (2018). Bacterial colonization in COPD patients admitted to a Rehabilitation Respiratory Unit and impact on length of Stay: a real-life study. COPD.

[9] Rollnik JD, Bertram M, Bucka C, Hartwich M, Jobges M, Ketter G, Leineweber B, Mertl-Rotzer M, Nowak DA, Platz T, Scheidtmann K, Thomas R, von Rosen F, Wallesch CW, Woldag H, Peschel P, Mehrholz J, Pohl M (2017). Outcome of neurological early rehabilitation patients carrying multi-drug resistant bacteria: results from a German multi-center study. BMC Neurol.

[10] Schonhofer B, Geiseler J, Dellweg D, Fuchs H, Moerer O, Weber-Carstens S, Westhoff M, Windisch W. Prolonged weaning: S2k Guideline published by the German respiratory society. Respiration 2020: 1–102. 10.1159/000510085 33302267

[11] Cerceo E, Deitelzweig SB, Sherman BM, Amin AN (2016). Multidrug-resistant gram-negative bacterial infections in the hospital setting: overview, implications for clinical practice, and Emerging Treatment options. Microb Drug Resist.

[12] Boles JM, Bion J, Connors A, Herridge M, Marsh B, Melot C, Pearl R, Silverman H, Stanchina M, Vieillard-Baron A, Welte T (2007). Weaning from mechanical ventilation. Eur Respir J.

[13] Schonhofer B, Geiseler J, Dellweg D, Moerer O, Barchfeld T, Fuchs H, Karg O, Rosseau S, Sitter H, Weber-Carstens S, Westhoff M, Windisch W (2015). S2k-Guideline prolonged Weaning. Pneumologie.

[14] Jubran A, Grant BJ, Duffner LA, Collins EG, Lanuza DM, Hoffman LA, Tobin MJ (2013). Effect of pressure support vs unassisted breathing through a tracheostomy collar on weaning duration in patients requiring prolonged mechanical ventilation: a randomized trial. JAMA.

[15] Levey AS, Stevens LA, Schmid CH, Zhang YL, Castro AF 3rd, Feldman HI, Kusek JW, Eggers P, Van Lente F, Greene T, Coresh J, Ckd EPI. A new equation to estimate glomerular filtration rate. Ann Intern Med. 2009;150(9):604–12. 10.7326/0003-4819-150-9-200905050-00006 PMC2763564 19414839

[16] Vardakas KZ, Rafailidis PI, Konstantelias AA, Falagas ME (2013). Predictors of mortality in patients with infections due to multi-drug resistant Gram negative bacteria: the study, the patient, the bug or the drug?. J Infect.

[17] Raman G, Avendano EE, Chan J, Merchant S, Puzniak L (2018). Risk factors for hospitalized patients with resistant or multidrug-resistant Pseudomonas aeruginosa infections: a systematic review and meta-analysis. Antimicrob Resist Infect Control.

[18] Bassetti M, Righi E, Vena A, Graziano E, Russo A, Peghin M (2018). Risk stratification and treatment of ICU-acquired pneumonia caused by multidrug- resistant/extensively drug-resistant/pandrug-resistant bacteria. Curr Opin Crit Care.

[19] Ceccarelli G, Alessandri F, Moretti S, Borsetti A, Maggiorella MT, Fabris S, Russo A, Ruberto F, De Meo D, Ciccozzi M, Mastroianni CM, Venditti M, Pugliese F, d’Ettorre G. Clinical Impact of Colonization with Carbapenem-Resistant Gram-Negative Bacteria in Critically Ill Patients Admitted for Severe Trauma. *Pathogens* 2022: 11(11). 10.3390/pathogens11111295 PMC9695038 36365046

[20] Grasselli G, Scaravilli V, Alagna L, Bombino M, De Falco S, Bandera A, Abbruzzese C, Patroniti N, Gori A, Pesenti A (2019). Gastrointestinal colonization with multidrug-resistant Gram-negative bacteria during extracorporeal membrane oxygenation: effect on the risk of subsequent infections and impact on patient outcome. Ann Intensive Care.

[21] Prado V, Hernandez-Tejero M, Mucke MM, Marco F, Gu W, Amoros A, Toapanta D, Reverter E, Pena-Ramirez C, Altenpeter L, Bassegoda O, Mezzano G, Aziz F, Juanola A, Rodriguez-Tajes S, Chamorro V, Lopez D, Reyes M, Hogardt M, Kempf VAJ, Ferstl PG, Zeuzem S, Martinez JA, Vila J, Arroyo V, Trebicka J, Fernandez J (2022). Rectal colonization by resistant bacteria increases the risk of infection by the colonizing strain in critically ill patients with cirrhosis. J Hepatol.

[22] van Prehn J, Kaiser AM, van der Werff SD, van Mansfeld R, Vandenbroucke-Grauls C (2018). Colonization sites in carriers of ESBL-producing Gram-negative bacteria. Antimicrob Resist Infect Control.

[23] Bickenbach J, Schoneis D, Marx G, Marx N, Lemmen S, Dreher M (2018). Impact of multidrug-resistant bacteria on outcome in patients with prolonged weaning. BMC Pulm Med.

[24] Magret M, Lisboa T, Martin-Loeches I, Manez R, Nauwynck M, Wrigge H, Cardellino S, Diaz E, Koulenti D, Rello J, Group E-VCS (2011). Bacteremia is an independent risk factor for mortality in nosocomial pneumonia: a prospective and observational multicenter study. Crit Care.

[25] Agbaht K, Diaz E, Munoz E, Lisboa T, Gomez F, Depuydt PO, Blot SI, Rello J (2007). Bacteremia in patients with ventilator-associated pneumonia is associated with increased mortality: a study comparing bacteremic vs. nonbacteremic ventilator-associated pneumonia. Crit Care Med.

[26] Callejo-Torre F, Eiros Bouza JM, Olaechea Astigarraga P, Coma Del Corral MJ, Palomar Martinez M, Alvarez-Lerma F, Lopez-Pueyo MJ (2016). Risk factors for methicillin-resistant Staphylococcus aureus colonisation or infection in intensive care units and their reliability for predicting MRSA on ICU admission. Infez Med.

[27] Kollef MH, Micek ST (2012). Antimicrobial stewardship programs: mandatory for all ICUs. Crit Care.

[28] Kollef MH, Bassetti M, Francois B, Burnham J, Dimopoulos G, Garnacho-Montero J, Lipman J, Luyt CE, Nicolau DP, Postma MJ, Torres A, Welte T, Wunderink RG. The intensive care medicine research agenda on multidrug-resistant bacteria, antibiotics, and stewardship. Intensive Care Med 2017: 43(9): 1187–97. 10.1007/s00134-017-4682-7 PMC6204331 28160023

[29] Charlson ME, Pompei P, Ales KL, MacKenzie CR (1987). A new method of classifying prognostic comorbidity in longitudinal studies: development and validation. J Chronic Dis.

